# A critique of narrative reviews of the evidence-base for ECT in depression

**DOI:** 10.1017/S2045796021000731

**Published:** 2022-01-27

**Authors:** C. F. Meechan, K. R. Laws, A. H. Young, D. M. McLoughlin, S. Jauhar

**Affiliations:** 1 Woodland View Hospital, Irvine, Ayrshire & Arran, UK; 2 School of Life and Medical Sciences, University of Hertfordshire, UK; 3 Centre for Affective Disorders, Psychological Medicine, IoPPN, King's College, London, UK; 4 Department of Psychiatry and Trinity College Institute of Neuroscience, Trinity College Dublin, St Patrick's University Hospital, Ireland

**Keywords:** Electroconvulsive therapy, meta-analysis, depression, evidence-based medicine

## Abstract

There has been recent debate regarding the efficacy of electroconvulsive therapy in the treatment of depression. This has been based on narrative reviews that contradict existing systematic reviews and meta-analyses. In this special article, we highlight the mistakes that occur when interpreting evidence using narrative reviews, as opposed to conventional systematic reviews and meta-analyses.

## Introduction

Electroconvulsive therapy (ECT) has been used in the treatment of major mental illness, particularly depression, since its initial recorded use in 1938 (Cerletti and Bini, 1938). Since then, significant improvements have emerged in its evidence-based practice (Kirov *et al*., 2021).

ECT has been evaluated and approved by the National Institute for Health and Care Excellence (NICE) in the UK (NICE, 2003), Food and Drug Administration (FDA) in the USA (FDA, 2018) and various international professional organisations (Bennabi *et al*., 2019; Malhi *et al*., 2020). Both NICE and FDA additionally incorporated specialist/professional and patient/carer group perspectives. Specifically, NICE used a systematic review commissioned by the Department of Health (Greenhalgh *et al*., 2005), based on the systematic review and meta-analysis by the UK ECT Group (UK ECT Review Group, 2003).

Despite this, misgivings and misunderstandings persist about both relative safety and efficacy of ECT. For example, a group of mental health professionals, patients and relatives recently wrote to the Chair of the Care Quality Commission (CQC) in the UK, stating: ‘Given the high risk of permanent memory loss and the small mortality risk, the longstanding failure to determine whether or not ECT works means that its use should be immediately suspended until a series of well designed, randomised, placebo controlled studies have investigated whether there really are any significant benefits against which the proven significant risks can be weighed’ (Read, 2020). Calls have also been made for an independent commission into ECT in the UK (Cunliffe and Johnstone, 2020).

Evidence cited to underpin these claims consists of a narrative review of the efficacy of ECT in depression (Read and Bentall, 2010) published in this journal, and a recent narrative review, which aimed to assess the quality of randomised controlled trials (RCTs) of ECT *v.* simulated ECT (sECT) for treatment of depression (Read *et al*., 2019). Although examining data from the same time period (no new placebo-controlled studies have been published in the interim), conclusions of both reviews oppose the findings and recommendations from NICE, FDA and multiple meta-analyses (Janicak *et al*., 1985; Kho *et al*., 2003; UK ECT Review Group, 2003; Pagnin *et al*., 2004; Greenhalgh *et al*., 2005; Gábor and László, 2005). Given this discrepancy and that these two narrative reviews provide the basis for recent clamour against ECT, we sought to examine these reviews.

## What is the evidence base for efficacy of ECT in the treatment of depression?

Briefly, clinical trial evidence for ECT in depression can be divided into original placebo-controlled (sECT) RCTs, further RCTs assessing ECT *v.* antidepressant, ECT *v.* repetitive transcranial magnetic stimulation (rTMS), and RCTs comparing different forms of ECT and pairwise and network meta-analyses of these trials.

Ten ECT-sECT RCTs for depression have been published (summarised in Table 1). In a systematic review and meta-analysis, the UK ECT Review Group (UK ECT Review Group, 2003) identified six relevant trials, demonstrating ECT was more effective than sECT with a large standardised effect size of −0.91 (95% CI −1.27 to −0.54). These findings are broadly consistent across relevant meta-analyses (summarised in Table 2). The UK ECT Review Group also found ECT superior to antidepressants for depression (effect size −0.80 [95% CI −1.29 to −0.29]).

**Table 1.** Original ECT *v.* sECT trials identified by Read and Bentall (2010)

Study (Year)	Relevant treatment arms	Primary outcome	Included by	Comments
Brill *et al*. (1959)	ECT (*N* = 21) *v*. sECT (*N* = 9) ECT subgroups: (a) ECT alone (*N* = 8) (b) ECT + succinylcholine (*N* = 10) (c) ECT + thiopental (*N* = 3) sECT subgroups: (a) Thiopental (*N* = 5) (b) Nitrous oxide (*N* = 4)	One month after the end of treatment (20 treatments) ECT non-significant percentage improvement 16/21 *v*. 4/9 Improvement was defined as a positive outcome in at least two of: (a) psychiatric evaluation (b) Lorr Psychiatric Rating Scale (c) psychological testing	*Pagnin* OR 3.824 (95% CI 0.761 to 19.204) *R & B* reported as non-significant	Experimental study designed to investigate the extent to which the following contributed to the therapeutic effectiveness of treatment with ECT: (1) passing an electrical current through the brain; (2) rapidly induced loss of consciousness; and (3) motor convulsion
Harris and Robin (1960)	ECT (*N* = 4) *v*. sECT (*N* = 4)	After 2 weeks (4 treatments), 2/4 ECT patients met the outcome of ‘great improvement’ compared with 0/4 in sECT group Differences were not statistically significant Outcome measure was ‘global assessments’ as defined in the paper	*Pagnin* OR 17.000 (95% CI 0.201 to 1437.8) *R & B* reported as non-significant	Open trial designed to assess effectiveness of phenelzine for depression. Contained arms including ECT + placebo (ECT) and anaesthesia + placebo (sECT) After 2 weeks, sECT arm were switched to receive ECT
Wilson *et al*. (1963)	ECT (*N* = 6) *v*. sECT (*N* = 6)	ECT more effective than sECT using HDRS	*UKECTRG* ECT more effective than sECT, not significant; SES −1.078 (95% CI −2.289 to 0.133) *R & B* reported as non-significant	Designed to compare ECT, imipramine, ECT + imipramine and sECT, with two of the four arms providing relevant data
Fahy *et al*. (1963)	ECT (*N* = 17) *v*. thiopentone (*N* = 17)	After 3 weeks (6 treatments) 6/17 ECT *v*. 2/17 thiopentone (sECT) were rated as ‘recovered’/‘minimal symptoms only’. Not statistically significant	*Pagnin* OR = 3.765 (95% CI 0.683 to 20.773) *R & B* reported as non-significant	Designed to compare ECT with imipramine, not sECT, but did contain an arm giving thiopentone providing relevant data
Freeman *et al*. (1978)	Bilateral ECT (*N* = 20) *v*. sECT (*N* = 20)	After 1 week (2 treatments) ECT significant improvement in HDRS	*UKECTRG* ECT more effective than sECT, not significant, SES -0.629 (95% CI −1.264 to 0.006) *R & B* reported as significant difference	After 1 week sECT arm switched to ECT as it was felt unethical to continue sECT
Lambourn and Gill (1978)	Unilateral ECT (*N* = 13) *v*. sECT (*N* = 13)	After 2 weeks (6 treatments) Wilcoxon test (1-tailed) no difference in HDRS (mean rank 23.12, *Z* = −0.31, *p* = 0.38)	*UKECTRG* ECT more effective than sECT but not significant. SES −0⋅170 (95% CI −0.940 to 0.600) *Pagnin* OR 1.000 (95% CI 0.245 to 4.084) *R & B* reported as non-significant	Double-blind RCT comparing unilateral ECT with sECT. Utilising unilateral rather than bilateral mode of ECT may have led to under-dosing in the treatment arm
Johnstone *et al*. (1980)	Bilateral ECT (*N* = 31) *v*. sECT (*N* = 31)	After 4 weeks (8 treatments), ECT significantly more effective than sECT using HDRS (*F*(_1,54_) = 7.8, *p* < 0.01)	*UKECTRG* ECT more effective than sECT; SES −0.739 (95% CI −1.253 to −0.224) *R & B* erroneously reported as significant difference during treatment in deluded but not agitated or retarded subgroups. No such data included or discussed in the study	Double-blind RCT comparing bilateral ECT with sECT
West (1981)	Bilateral ECT (*N* = 11) *v*. sECT (*N* = 11)	After 3 weeks (6 treatments) ECT significantly more effective than sECT across all three outcomes; psychiatrists' visual analogue rating scale, nurses' 9-point rating scale and BDI	*UKECTRG* ECT more effective than sECT, SES −1.255 (95% CI −2.170 to −0.341) *Pagnin* OR 86.100 (95% CI 5.409 to 1370.5) *R & B* reported significant difference during treatment	Double-blind RCT comparing bilateral ECT with sECT
Brandon *et al*. (1984)	Bilateral ECT (*N* = 53) *v*. sECT (*N* = 42) Subgroups of depression with retardation, delusions and neurosis	After 4 weeks (8 treatments) ECT showed a significant improvement over sECT on HDRS in the ‘combined depression’ group	*Pagnin* OR 2.162 (95% CI 0.870 to 5.374) *R & B* reported as significant difference for ‘deluded’ and ‘retarded’ subgroups	Double-blind RCT comparing bilateral ECT with sECT
Gregory *et al*. (1985)	Unilateral ECT (*N* = 19) *v*. bilateral ECT (*N* = 21) *v*. sECT (*N* = 20) 6 treatment sessions	After 3 weeks (6 treatments) unilateral and bilateral ECT significantly more effective than sECT on MADRS, HDRS and PIRS	*UKECTRG* ECT more effective than sECT, SES −1.418 (95% CI −2.012 to −0.824) *R & B* reported as significant difference	Double-blind RCT comparing bilateral ECT, unilateral ECT and sECT

BDI, Beck Depression Inventory; HDRS, Hamilton Depression Rating Scale; MADRS, Montgomery Åsberg Depression Rating Scale; MPRS, Malamud Psychiatric Rating Scale; PIRS, Psychological Impairments Rating Scale; CI, confidence interval; SES, standardised effect size.

Pagnin, Pagnin *et al*. (2004); R & B, Read and Bentall (2010); UKECTRG, UK ECT Review Group (2003).

**Table 2.** Meta-analyses of ECT *v.* sECT studies

ECT *v.* sECT meta-analysis	Number of studies	Number of participants	Outcome
Janicak *et al*. (1985)	6	205	ECT significantly superior to sECT (*χ*^2^ = 21.54, df = 1, *p* < 0.001) and 32% more effective (Mantel–Haenszel statistic)
Kho *et al*. (2003)	2	106	ECT significantly superior compared with sECT. Mean effect size 0.95 (0.35–1.54) *p* < 0.05
UK ECT Review Group (2003)	6	256	ECT significantly more effective than sECT. Standardised effect size −0.91 (95% CI −1.27 to −0.54)
Pagnin *et al*. (2004)	7	245	ECT significantly more effective than sECT (association *χ*^2^ = 6.87, df = 1, *p* = 0.0087). Response 3 times greater than sECT (OR 2.83, 95% CI 1.30 to 6.17)
Gabor and Laszlo (2005)	9	321	ECT significantly superior to sECT (*χ*^2^ = 10.506, df = 1, *p* = 0.0012)
Greenhalgh *et al*. (2005)	1	70	ECT significantly more effective than sECT (RR = 1.98, 95% CI 1.05 to 3.73, *p* = 0.03)

The original ECT-sECT RCTs, conducted between the 1950s and mid-1980s, would not meet contemporary standards of evidence-based medicine (EBM) (as is the case for the majority of medical trials from that era). Numerous additional modern RCTs have assessed the efficacy of ECT against non-pharmacological interventions such as rTMS. In a systematic review and meta-analysis comparing ECT with rTMS (*n* = 294), remission rates were significantly higher for ECT *v.* rTMS (52 *v*. 34% respectively, OR = 0.46 [95% CI: 0.22 to 0.96; z = −2.06; p =0.04]) with a significantly more pronounced reduction of depressive symptoms in the ECT group (Hedges' *g* = −0.93; *p* = 0.007) (Berlim *et al*., 2013). A subsequent meta-analysis (*n* = 429) (Ren *et al*., 2014) found ECT superior to high-frequency rTMS in response (64.4 *v*. 48.7%, RR = 1.41, *p* = 0.03) and remission (52.9 *v*. 33.6%, RR = 1.38, *p* = 0.006). Neither review found a significant difference in discontinuation between groups. Whilst this illustrates improved trial design (contemporary ECT modes and active comparators), these trials usually lack blinding of participants.

Andrade (Andrade, 2021) gives an example of three RCTs of ECT *v*. active comparators (Sackeim et al., 1987, 1993, 2000) that demonstrate the efficacy of bilateral ECT and high-dose right unilateral ECT in people with Major Depressive Disorder (MDD) compared to low-dose right unilateral ECT. Meta-analytic data also demonstrate the efficacy of brief-pulse right unilateral ECT compared to ultra-brief pulse right unilateral ECT (standardised mean difference = 0.25 [95% CI 0.08 to 0.41]; *p* = 0.004) (Tor *et al*., 2015). As Andrade (2021) points out, high-quality RCTs, utilising contemporary ECT techniques demonstrate the efficacy of ECT when tested blindly against an active comparator (in the form of an alternative mode of ECT).

Thus, the evidence base for ECT consists of RCTs providing converging evidence consistently demonstrating greater efficacy than numerous comparators, including sECT, pharmacological and non-pharmacological interventions as well as alternative modes of ECT.

## Read and Bentall (2010)

In their narrative review, Read and Bentall identified ten studies from 1959 to 1985 comparing ECT with sECT and eight meta-analyses for ECT compared with sECT, in depression. In addition to summarising these studies, they covered potential side-effects, including what they describe as ‘brain damage’. They report ‘…placebo-controlled studies show minimal support for effectiveness with either depression or “schizophrenia”…’ and conclude, ‘Given the strong evidence (summarised here) of persistent and, for some, permanent brain dysfunction, primarily evidenced in the form of retrograde and anterograde amnesia, and the evidence of a slight but significant increased risk of death, the cost-benefit analysis for ECT is so poor that its use cannot be scientifically justified.’

These bold claims directly contradict evidence available at the time, as well as recently published reviews (Mutz *et al*., 2019; Andrade, 2021; Kirov *et al*., 2021). Examining this review from an evidence-based perspective highlights common problems in narrative as opposed to systematic reviews.

## Lack of an effect size measure, i.e. meta-analysis

RCTs included in the Read and Bentall review are summarised in Table 1, which also indicates inclusion in either of the two major meta-analyses available before 2010 from the UK ECT Review Group (UK ECT Review Group, 2003) and Pagnin *et al*. (Pagnin *et al.*, 2004). A number of the historical studies comparing ECT to sECT were methodologically flawed and, therefore, only six of the ten were included in the comprehensive systematic review by the UK ECT Group. No clear study inclusion criteria are provided in Read and Bentall's narrative review.

Table 1 (p. 336) states only five of ten studies were statistically significant. Such vote-counting is a flawed method of research synthesis, and acknowledged as misleading (see Hedges and Olkin, 1980; Gurevitch *et al*., 2018) – not least of all because it does not quantify the magnitude of group differences or consider sample sizes. One study (Harris and Robin, 1960) only included four patients in each arm (eight in total), another (Wilson *et al*., 1963) only 6 patients (12 in total); therefore, statistical significance of the individual study does not necessarily convey useful information. In some of these studies (e.g. Brill *et al*., 1959; Harris and Robin, 1960), ECT showed greater efficacy than comparator, though not significantly so. When seven of the studies included in the review were meta-analysed by Pagnin et al (Pagnin *et al.,* 2004) six years earlier, a statistically significant superiority for ECT over sECT was seen (see Table 2). A related point is selective citing of follow-up data, and conclusions drawn, when studies were not conducted to test longer-term effects and had naturalistic designs following acute treatment, i.e. were not conducted under RCT conditions.

**Fig. 1.**
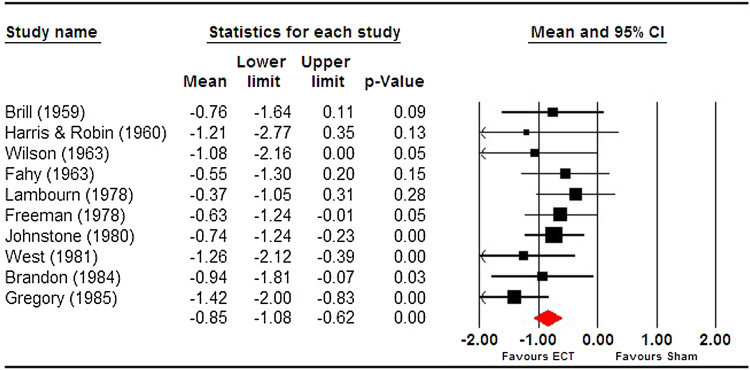
Meta-analysis of ECT vs sECT trials for depression, included in Read and Bentall (2010).

Given the lack of reporting of an effect size across all included trials, we conducted our own meta-analysis of trials included in the narrative review. Read and Bentall suggested Ulett *et al*. (1956) be excluded as the sample would not meet the criteria for depression diagnosis and we therefore excluded it. We found a pooled effect size that was both large and significant (*g* = −0.85 [95% CI −1.08 to −0.63]) (see Fig. 1). Heterogeneity was extremely low (*I*^2^ = 0) and non-significant (*Q* = 8.96, df = 9, *p* = 0.44). This effect size is comparable to that reported by the UK ECT group, −0.91 (95% CI −1.27 to −0.54) derived from six trials.

## Selective citing/reporting

In their discussion of ECT's effects on memory, Read and Bentall (2010) and Read *et al*. (2019) both rely on a single systematic review of patient perspectives on ECT (Rose *et al*., 2003) that included seven studies measuring subjective memory impairment as evidence of memory dysfunction. We note this review has been criticised (Bergsholm, 2012), on account of included studies. The review stated the inclusion of reports from at least 6 months post-ECT, but three of the seven studies failed to meet this criterion – obtaining patient self-report much sooner, e.g. Pettinati *et al*. (1994) at <48 h after ECT. It should be noted that Rose *et al*. did not report control data, when it is known that self-reported memory problems also occur in sham ECT and some research shows that such problems might be indistinguishable from those in real ECT (Frith *et al*., 1983). Indeed, Frith *et al*. argue that ‘…complaints about the memory problems seem not to be associated with real ECT, but with a relatively high degree of depression and an unsuccessful treatment’ (p. 615), and cognitive impairment in depression is recognised in MDD (Rock *et al*., 2014).

In contrast to Rose *et al*. in a 2019 systematic review (Jones and McCollum, 2019), out of seven subjective memory studies identified and conducted prior to the Read and Bentall (2010) review, four found improved subjective memory scores (Coleman *et al*., 1996; Ng *et al*., 2000; Schulze-Rauschenbach *et al*., 2005; Arts *et al*., 2006), two reported no difference (Frith *et al*., 1983; Berman *et al*., 2008), only one showed reduced subjective memory scores during ECT, in a mixed sample of people with mania, schizophrenia and depression, noting normalisation at follow-up (Ikeji *et al*., 1999). While Read and Bentall (2010) selectively cite additional studies regarding retrograde and anterograde amnesia, they do not include the six subjective memory studies that demonstrated views contrary to their own, despite all being available at the time.

In a meta-analysis on objective cognitive performance associated with ECT reported in the same year and including 84 studies (75 of which included memory domains) (Semkovska and McLoughlin, 2010) found significant decreases in cognitive performance occurred 0–3 days post-ECT in 72% of 24 cognitive variables (effect sizes ranging from −1.10 [95% CI −1.53 to −0.67] to −0.21 [95% CI −0.40 to 0.01]). Fifteen days post-ECT there were no negative effect sizes and 57% of variables showed a positive effect size (improved cognition), ranging from 0.35 [95% CI 0.07 to 0.63] to 0.75 [95% CI 0.43 to 1.08]. Read and Bentall (2010) only included one of the 75 objective memory studies (Neylan *et al*., 2001) despite being available at the time. Read and Bentall (2010) defend the use of subjective memory scores, but do not provide a representative summary of evidence underpinning such scores or methodological issues surrounding comparison between subjective and objective measures of cognitive performance in depression.

A related point is ‘cherry-picking’ of outcome measures. For example, the Northwick Park trial's primary outcome measure was the Hamilton Depression Rating Scale (HDRS); the study was powered based on this measure, which showed a statistically significant improved outcome with real ECT (Johnstone *et al*., 1980). Rather than focusing on the primary outcome and this significant difference, the authors highlight secondary outcomes that failed to show statistical significance.

Utilising the widely used AMSTAR 2 tool (Shea *et al*., 2017) to assess the quality of reviews, we found Read and Bentall (2010) scored ‘critically low’ in quality. Analyses were independently conducted by two authors (CM and KL: see online Supplementary Table 3). Inter-rater reliability was 93.75% agreement and Cohen's *k* = 0.82 which indicates ‘almost perfect agreement’ (Landis and Koch, 1977).

## Read, Kirsch and McGrath Review (2019)

Read *et al*. (2019) aimed to assess the quality of ECT *v*. sECT RCTs and related meta-analyses. Eleven original RCTs and five meta-analyses were identified. They used their own un-validated 24-point quality scale to evaluate trials and commented on linked meta-analyses.

They reported variation in number of original studies included across meta-analyses, ranging from 1 to 7 and ‘little attention’ paid to the limitations of the original studies. The mean ‘quality score’ of original RCTs was 12.3 out of 24. The authors dedicated a substantial portion of the review to side-effects.

As with Read and Bentall (2010), Read *et al*. (2019) contains major methodological shortcomings related to EBM. Specific concerns include:

## The review was not systematic and ‘critically low’ in quality

Read *et al*. (2019) failed to pre-register a protocol for their review. An essential feature of any high-quality systematic review is publication of a pre-registered protocol that identifies main objectives, key design features and planned analyses, inclusion/exclusion criteria, and both promote transparency and reduce potential bias. Read *et al*. (2019) scored ‘critically low’ when assessed using the AMSTAR 2 tool (Shea *et al*., 2017). Analyses were independently conducted by two authors (CM and KL). Inter-rater reliability was 100% agreement (perfect agreement) and Cohen's *k* = 1 (Landis and Koch, 1977) (see online Supplementary Table 3). According to AMSTAR 2 guidance, reviews that receive a ‘critically low’ ratings (i.e. both Read and Bentall (2010) and Read *et al*. (2019) means that ‘The review has more than one critical flaw and should not be relied on to provide an accurate and comprehensive summary of the available studies’.

## Use of an un-validated quality scale, biased in favour of inflating poor-quality ratings

The authors combined the aspects of the Cochrane Risk of Bias tool with their own measures to assess the quality of ECT-sECT RCTs. The use of any quality scale itself is problematic, emphasised in the original paper from which ‘risk of bias’ domains were taken (Higgins *et al*., 2011). Cochrane advance seven principles for assessing risk of bias, the first of which states: ‘Do not use quality scales’. The inclusion by Read *et al*., (2019) of items within the scale such as ‘patient ratings’ and ‘suicide measure’ was weighted the same as randomisation and blinding. While the authors tried to account for this by including multiple items for domains such as randomisation, blinding and diagnosis, the result is a scale that allows for inconsistent, internally invalid scores. Read *et al*. (2019) claimed to employ risk of bias domains derived from the Cochrane RoB tool (randomisation, blinding, incomplete outcome data and selective reporting). These domains (from the older RoB tool that Cochrane initially allowed while piloting the newer RoB2 tool) are used in ways not advocated by Cochrane. In direct conflict with Cochrane guidance, Read *et al*. (2019) collapse the Cochrane ‘No’ and ‘Unclear’ categories into a bimodal distinction where ‘No’ meant either no evidence OR clear negative evidence. Cochrane advice is that ‘A judgment of unclear risk should also be made if what happened in the trial is known but the associated risk of bias is unknown’. This re-scaling artificially inflates ‘No’ responses. To take a specific example, for the item ‘Decliners described’ (i.e. any description of people who were approached, but declined to participate), multiple studies are negatively scored because they do not report on any decliners (e.g. West, 1981; Lambourn and Gill, 1978). Under Cochrane guidance, the appropriate response is ‘unclear’. The inclusion by Read *et al*. of a quality-of-life scale such as HoNOS is difficult to understand, given HoNOS was not developed until several years after the most recent ECT-sECT trial in the early 1990s (Wing *et al*., 1998). The same would be true for requiring a ‘validated depression scale for some of the included studies, e.g., Hamilton, Montgomery, Beck’. Several studies were conducted prior to the development of any of these scales – the earliest to be developed would appear to be Hamilton (1960).

Similarly, for other items, the scoring and scaling introduces further biases against accurate reporting of quality. For example, pooling of age and gender into a single criterion is baffling – these two features are independent. The scaling used means failing on either one alone is a failing on both and therefore biases against fair quality assessment. The authors provide no cut-off scores for their scale and thus no criterion demonstrating what would constitute a good or poor quality trial. Regardless of the multiple issues outlined, reporting a mean score of 12.3 out of 24 is uninterpretable. We have already seen that Read *et al*. have used the Cochrane RoB scale against advice; however, Cochrane are also clear that ‘The use of scales (in which scores for multiple items are added up to produce a total) is discouraged’. The failure to employ any standardised assessment of quality critically undermines both any objectivity and ability to draw conclusions about quality – especially as many such scales exist that are standardised, reliable and widely employed. As noted decades ago, Greenland (1994) referred to the practice of quality scoring as ‘Perhaps the most insidious form of subjectivity masquerading as objectivity’ (p. 295). Given the above limitations, we assessed risk of bias for original ECT-sECT RCTs using the Cochrane RoB2 tool (Sterne *et al*., 2019), see online Supplementary Fig. 2, Table 4 and Table 5.

## Effect sizes and study quality

Meta-analyses of small underpowered trials can exaggerate effect sizes (Dechartres *et al*., 2013). While some individual simulated *v.* real ECT RCTs were adequately powered (Johnstone *et al*., 1980; Brandon *et al*., 1984) and demonstrated a significant effect, several have small sample sizes. Nevertheless, we found little evidence of a small study effect in terms of publication bias – Egger's test (intercept −0.75, *p* = 0.59) and Begg and Mazumdar Kendall's *τ* (−0.24, *p* = 0.33) were non-significant. The funnel plot however showed some visual asymmetry and a trim and fill analysis suggested three potentially missing studies – adjusting the effect size downward from −0.85 to −0.68.

Importantly, meta-regression showed the Read *et al*. (2019) quality scores were not significantly related to effect sizes (*Z* = 1.31, *p* = 0.19) (see online Supplementary Fig. 3). This has implications for their general criticism of past meta-analyses, where they argue, ‘All five of the meta-analyses claim that ECT is effective for depression but, as we have seen, they are all of a poor standard, not least because none of them pay sufficient attention to the quality of the papers on which they base this claim.’ The current analysis suggests at least two possibilities: either study quality is not a key driver of effect size in sECT trials or the Read *et al*. assessment of study quality fails to capture relevant quality differences.

## Blinding and the placebo effect

Read *et al*. (2019) question the integrity of RCTs being ‘double blind’ on the assumption that the involvement of patients who had previously received ECT renders them ‘unblinded’ ‘because they know that ECT is always followed by headaches and disorientation’ (p. 89). They conclude ‘by not excluding people who have previously had ECT all 11 studies exaggerated the difference between ECT and SECT in ECT's favor, and that none were truly blind studies’ (p. 97). As pointed out by Anderson (Anderson 2021), this is itself a weak argument *a priori*, since side-effects such as headache and temporary confusion are common in general anaesthesia, and moreover, the claim ‘…is contradicted by audit figures (Scottish ECT Accreditation Network 2019) showing the incidence of post-ECT headache to be about 30% and confusion about 20%’ (Anderson, 2021).

Despite this, we conducted sub-group analysis to assess whether effect size was affected by the experience of previous ECT. Read *et al*. (2019) identified five trials that included people who had received ECT previously (Brill *et al*., 1959; Freeman *et al*., 1978; Lambourn and Gill, 1978; Johnstone *et al*., 1980; Brandon *et al*., 1984) and five where previous ECT was unascertained (Harris and Robin, 1960; Fahy *et al*., 1963; Wilson *et al*., 1963; West, 1981; Gregory *et al*., 1985) – note Read *et al*. (2019) collapsed both groups suggesting ‘None of the participants had had ECT at any time prior to the study’. Contrary to Read *et al*., (2019), the pooled effect size was significantly larger (*Q* = 4.01, *p* < 0.05) in trials where previous ECT was unknown or unreported (−1.13 [95% CI −1.50 to −0.76]) rather than those reporting previous treatment with ECT (−0.67 [95% CI −0.97 to −0.37]) (see online Supplementary Fig. 3). Hence, trials where patients previously received ECT did not bias in favour of the ECT arm through patients being ‘unblinded’. It is also worth noting that significant differences demonstrated when comparing different modes of ECT in contemporary trials (Sackeim et al., 1987, 1993, 2000) would not be found if ECT was simply a placebo response.

## Misunderstanding the nature of EBM, placebo and active comparators

What of the authors' claim that the quality of the sECT-ECT studies is ‘so poor’ that they have no utility? One issue relates to the difference between identifying study limitations, contrasted with demonstrating study quality is ‘so poor’ that it should be discarded. EBM has developed over the last 50 years, such that many, if not most, trials from this era (including sECT-ECT studies) would likely not be consistent with contemporary standards yet are still of high enough standard to contribute to the evidence base. Second, it represents a misunderstanding of principles of EBM that both Read and Bentall (2010) and Read *et al*. (2019) focus exclusively on ECT-sECT RCTs and their associated meta-analyses as the *only* relevant evidence. The authors fail to consider these studies within the wider context of other relevant evidence.

Read *et al*., (2019) fail to comment on evidence from studies that demonstrate ECT's superiority to pharmacotherapy (e.g. Pagnin *et al*., 2004, UK ECT Review Group, 2003) and non-pharmacological biological treatments both directly (Ren *et al*., 2014) and indirectly (Mutz *et al*., 2019). Moreover, where available, active comparators, not placebo controls, are the best treatments to include in comparison arms, as these can produce real side-effects, thereby maximising any placebo or nocebo effect (Stafford *et al*., 2009). As pointed out by Andrade (Andrade, 2021), there are ‘large, well-designed, well-conducted, and well-analyzed modern era RCTs that show that bilateral and high dose right unilateral ECT are more effective than low dose right unilateral ECT, or that brief-pulse ECT is more effective than ultrabrief-pulse ECT’, with the latter providing an active comparator superior to that of placebo sECT. In other words, the ECT-sECT RCTs exist in the context of an extensive, robust and converging evidence base.

## Conclusion

Recent calls for banning ECT are based on selective narrative reviews written by authors who suggest ECT does not have efficacy *v.* sECT and has significant effects on patient safety that merit a public enquiry. Examining their evidence, we have identified numerous substantial problems that stem from these narrative reviews having inherent biases and major methodological shortcomings. We would suggest those concerned with interpreting evidence continue to use conventional standardised methods of systematic review and meta-analysis where possible and that policy decisions must continue to be based on this level of evidence.

Whilst we cannot agree with most of Read *et al*.'s empirical arguments, moving beyond disagreement is crucial. We therefore advocate for modern trials to optimise ECT side-effect monitoring, and studies to elucidate the mechanism of action of one of the most effective treatments we have in Psychiatry.

## Supplementary material

For supplementary material accompanying this paper visit https://doi.org/10.1017/S2045796021000731. click here to view supplementary material
