# Supplementary material for: A critique of narrative reviews of the evidence-base for ECT in depression
Source: Epidemiol Psychiatr Sci. 2022 Jan 27;31:e10. doi: 10.1017/S2045796021000731 (PMC8851059; doi:10.1017/S2045796021000731)
Supplement: Supplementary file 1 [file S2045796021000731sup001.docx]

**Supplementary Material**

***Table 3 AMSTAR2 ratings of Read and Bentall (2010) and Read et al (2019)***

|  | **Read & Bentall 2010** | | **Read et al 2019** | |
| --- | --- | --- | --- | --- |
|  | **CM** | **KL** | **CM** | **KL** |
| **Did the research questions and inclusion criteria for the review include the components of PICO?** | Y | Y | Y | Y |
| **Did the report of the review contain an explicit statement that the review methods were established prior to the conduct of the review and did the report justify any significant deviations from the protocol?** | N | N | N | N |
| **Did the review authors explain their selection of the study designs for inclusion in the review?** | N | Y | Y | Y |
| **Did the review authors use a comprehensive literature search strategy?** | PY | PY | N | N |
| **Did the review authors perform study selection in duplicate?** | N | N | N | N |
| **Did the review authors perform data extraction in duplicate?** | N | N | N | N |
| **Did the review authors provide a list of excluded studies and justify the exclusions?** | N | N | N | N |
| **Did the review authors describe the included studies in adequate detail?** | N | N | Y | Y |
| **Did the review authors use a satisfactory technique for assessing the risk of bias (RoB) in individual studies that were included in the review?** | N | N | N | N |
| **Did the review authors report on the sources of funding for the studies included in the review?** | N | N | N | N |
| **If meta-analysis was performed did the review authors use appropriate methods for statistical combination of results?** | NA | NA | NA | NA |
| **If meta-analysis was performed, did the review authors assess the potential impact of RoB in individual studies on the results of the meta-analysis or other evidence synthesis?** | NA | NA | NA | NA |
| **Did the review authors account for RoB in individual studies when interpreting/ discussing the results of the review?** | N | N | Y | Y |
| **Did the review authors provide a satisfactory explanation for, and discussion of, any heterogeneity observed in the results of the review?** | N | N | N | N |
| **If they performed quantitative synthesis did the review authors carry out an adequate investigation of publication bias (small study bias) and discuss its likely impact on the results of the review?** | NA | NA | NA | NA |
| **Did the review authors report any potential sources of conflict of interest, including any funding they received for conducting the review?** | Y | Y | Y | Y |
| **Outcome** | CRITICALLY LOW | CRITICALLY LOW | CRITICALLY LOW | CRITICALLY LOW |

**Figure 2 Risk of Bias for ECT-sECT RCTs**

**(Text to accompany Figure 2, Tables 4 & 5)**

We assessed the risk of bias for the original ECT-sECT RCTs using the Cochrane RoB2 tool (Sterne et al., 2019). Risk of bias was analysed by CM and scores subsequently discussed and refined by CM, KL and SJ. The results are presented in Figure 2. Domain 3 ‘missing outcome data’ and Domain 4 ‘Measurement of the outcome’ are both at relatively ‘low risk’. Domain 1 ‘randomisation process’ and Domain 5 ‘selection of the reported result’ show ‘some concerns’ and Domain 2 ‘deviations from intended interventions’ was particularly weak (see Table 4).

Concerning Domain 2, two specific sub-domains (2.2 and 2.5) were largely responsible for the overall score of ‘high risk’ of bias. Sub-domain 2.2 asks if those delivering the intervention were aware of the participants’ assigned intervention. Given the nature of administrating ECT all studies scored poorly here, as would any RCT involving an intervention where those administering cannot be blinded due to the nature of the intervention, such as surgery or psychotherapy. Importantly, there were no concerns regarding the blinding of those rating outcomes, with the exception of Wilson et al., (1963) (which is addressed in Domain 4).

The second sub-domain scoring poorly was domain 2.5 which asked if there was “non-adherence to the assigned intervention regimen that could have affected participants’ outcomes?” We investigated the direction of risk of bias for the seven studies scoring poorly in this domain (see Table 5). For four of the studies (Brill et al., 1959, Harris & Robin, 1960, Lambourn & Gill, 1978 and West, 1981) the direction of bias could not be ascertained due to a lack of information – in other words the high risk of bias could have favoured the sECT arm, the ECT arm or a combination of both. For the remaining three studies (Johnstone et al., 1980, Brandon et al., 1984 and Gregory et al., 1985), the direction of bias favoured the sECT arm *not* the ECT arm. For example, in Johnstone et al., 1980, one participant was withdrawn from the ECT arm for ‘poor response’ compared with three participants in the sECT arm, thus potentially deflating the response in the ECT arm compared to sECT. In summary, although seven studies score poorly for domain 2.5 (which largely drives the overall ‘high risk’ score for domain 2), no evidence suggests that these studies were biased in favour of ECT and some evidence suggested for at least three of them, the risk of bias favoured sECT.

It is worth highlighting that whilst Fahy et al., (1963) did not score poorly on domain 2.5 they did acquire an overall score of ‘high risk’ for Domain 2. This was because this was the only study in which participants were not blinded. Of relevance, however, is this that study was ‘non-significant’ and furthermore, patients were not aware they were receiving placebo but rather that “…the sleep injection was a complete treatment in its own right” thus making any effects of unblinding subject to the patients’ prior beliefs about ECT compared with a new ‘sleep injection treatment’ which remain unknown.

***Table 4 Risk of Bias domain scores for original ECT-sECT RCTs***

| **Study (year**) | **Domain 1 Randomisation  process** | **Domain 2 Deviations from the intended interventions** | **Domain 3 Missing outcome data** | **Domain 4 Measurement of the outcome** | **Domain 5 Selection of the reported result** | **Overall ROB** |
| --- | --- | --- | --- | --- | --- | --- |
| Brill *et al.*, (1959) | Some Concerns | High Risk | Low Risk | High Risk | Some Concerns | High Risk |
| Harris and Robin, (1960) | Low Risk | High Risk | Low Risk | High Risk | Some Concerns | High Risk |
| Wilson *et al.*, (1963) | Some Concerns | Low Risk | Low Risk | Some Concerns | Some Concerns | Some Concerns |
| Fahy *et al.,* (1963) | Some Concerns | High Risk | Low Risk | High Risk | Some Concerns | High Risk |
| Freeman *et al*., (1978) | Some Concerns | Low Risk | Low Risk | Low Risk | Some Concerns | Some Concerns |
| Lambourn and Gill, (1978) | Some Concerns | High Risk | Low Risk | Low Risk | High Risk | High Risk |
| Johnstone *et al.*, (1980) | Low Risk | High Risk | Low Risk | Low Risk | Some Concerns | High Risk |
| West (1981) | Some Concerns | High Risk | Low Risk | Low Risk | Some Concerns | High Risk |
| Brandon *et al.*, (1984) | Low Risk | High Risk | High Risk | Low Risk | Some Concerns | High Risk |
| Gregory *et al.,* (1985) | Some Concerns | High Risk | Low Risk | Low Risk | Some Concerns | High Risk |

***Table 5 Direction of Risk of Bias in sub-domain 2.5***

|  |  | **Reasons for withdrawal** | | |  |
| --- | --- | --- | --- | --- | --- |
| **Study (year)** | **Total withdrawn** | **Poor response** | **Got better** | **Other** | **Direction of Risk of Bias** |
| Brill *et al.,* (1959) | 45 | No Info | No Info | 45 cases were started but were dropped for a number of reasons, such as “pre-treatment improvement, refusal of treatment, elopement, etc”. No information on specific arms. | Risk of bias direction unknown |
| Harris & Robin (1960) | 4 | Nil | Nil | “physical symptoms unrelated to treatment”:- ECT (n=2), sECT (n=1)   “discharged herself” (Reason unknown) - ECT (n=0), sECT (n=1) | Risk of bias direction unknown |
| Lambourn & Gill (1978) | 6 | No Info | No Info | 6 patients were "lost from study" 3 in each arm (Reasons unknown) | Risk of bias direction unknown |
| Johnstone *et al.,* (1980) | 8 | ECT (n=1) sECT (n=3) | Nil | "minor vascular incident" ECT (n=1), sECT (n=0)  "withdrew consent" ECT (n=1), sECT (n=1)  "became manic" ECT (n=1), sECT (n=0) | Risk of bias toward sECT |
| West (1981) | 3 | ECT (n=1) sECT (n=1) | Nil | "could not complete BDI" ECT (n=1), sECT (n=0) | Risk of bias direction unknown |
| Brandon *et al.,* (1984) | 18 | ECT (n=1)  sECT (n=3) | Nil | "withdrew due to suicide" - ECT (n=0), sECT (n=1)   "Physical illness" - ECT (n = 1), sECT (n = 0)  "Self discharge" - ECT (n = 4), sECT (n = 5)  "Consent withdrawn" - ECT (n = 0), sECT (n = 1)  "Unknown" - ECT (n=0), sECT (n=2) | Risk of bias toward sECT |
| Gregory *et al*., (1985) | 25 | sECT  (n = 7) Uni-ECT (n = 5) Bi-ECT (n = 3) | sECT (n=0),  Uni-ECT (n=1),  Bi-ECT (n=4), | sECT – “Detained” (n = 1) Uni-ECT – “Withdrew consent” (n = 1) Bi-ECT – “Withdrew consent” (n=2) | Risk of Bias toward sECT* |

*Note there was a discrepancy in the numbers for Gregory et al., (1985). Forty-four patients remained after loss of 25 withdrawals. But complete data was provided for 60 patients. Total number of reasons for withdrawals given was 24 but 25 withdrawals were reported.

***Figure 3 Effect sizes for ECT-sECT RCTs including reported Vs unreported previous ECT treatment***


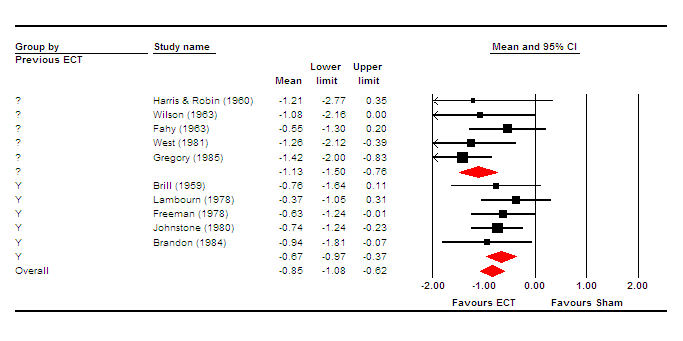
 Note Y = some participants had previously experienced ECT; ? = previous ECT was unreported

**References**

**Brandon, S., Cowley, P., McDonald, C., Neville, P., Palmer, R. and Wellstood-Eason, S**., 1984. Electroconvulsive therapy: results in depressive illness from the Leicestershire trial. *BMJ (Clinical Research Ed.)*, *288*(6410), pp.22-25.

**Brill, N.Q., Crumpton, E., Eiduson, S., Grayson, H.M., Hellman, L.I. and Richards, R.A.**, (1959). Relative effectiveness of various components of electroconvulsive therapy: an experimental study. *AMA Archives of Neurology & Psychiatry*, *81*(5), pp.627-635.

**Fahy, P., Imlah, N. and Harrington, J.** (1963) ‘A controlled comparison of electroconvulsive therapy, imipramine and thiropentone sleep in dperession. *Journal of Neuropsychiatry*, 4, pp. 310–314.

**Freeman, C.P.L., Basson, J.V. and Crighton, A.,** (1978). Double-blind controlled trial of electroconvulsive therapy (ECT) and simulated ECT in depressive illness. *The Lancet*, *311*(8067), pp.738-740.

**Gregory, S., Shawcross, C.R. and Gill, D.** (1985) ‘The Nottingham ECT Study. A double-blind comparison of bilateral, unilateral and simulated ECT in depressive illness’, *The British Journal of Psychiatry*, 146, pp. 520–524.

**Harris, J.A. and Robin, A.A.** (1960) ‘A controlled trial of phenelzine in depressive reactions’, *The Journal of Mental Science*, 106, pp. 1432–1437.

Johnstone, E.C. *et al.* (1980) ‘The Northwick Park electroconvulsive therapy trial’, *Lancet (London, England)*, 2(8208–8209), pp. 1317–1320.

**Lambourn, J. and Gill, D.** (1978) ‘A controlled comparison of simulated and real ECT’, *The British Journal of Psychiatry*, 133, pp. 514–519.

**Sterne, J.A., Savović, J., Page, M.J., Elbers, R.G., Blencowe, N.S., Boutron, I., Cates, C.J., Cheng, H.Y., Corbett, M.S., Eldridge, S.M. and Emberson, J.R.,** 2019. RoB 2: a revised tool for assessing risk of bias in randomised trials. *BMJ (Clinical Research ed.)*, 366l4898.

**West, E.D.** (1981) ‘Electric convulsion therapy in depression: a double-blind controlled trial.’, *BMJ (Clinical research ed.)*, 282(6261), pp. 355–357.

**Wilson, I.C., Vernon, J.T., Sandifer, M.G., Jr, and Guin, T.,** 1963. A controlled study of treatments of depression. *Journal of Neuropsychiatry*, 4, pp.331–337.
